# Fluorescence use in minimally invasive metabolic and bariatric surgery — a systematic review of the literature

**DOI:** 10.1007/s00423-023-02955-9

**Published:** 2023-05-30

**Authors:** Mateusz Wityk, Natalia Dowgiałło-Gornowicz, Igor Feszak, Maciej Bobowicz

**Affiliations:** 1 Department of General and Oncological Surgery, Voivodeship Specialist Hospital, 1 Hubalczykow Str. 76-200, Slupsk, Poland; 2 Department of General, Minimally Invasive and Elderly Surgery, University of Warmia and Mazury in Olsztyn, Niepodleglosci 44 Str., 10-045 Olsztyn, Poland; 3 Department of Radiology, Medical University of Gdansk, 17 Smoluchowskiego Str., 80-211 Gdansk, Poland

**Keywords:** ICG, Indocyanine green, Fluorescence, Metabolic and bariatric surgery

## Abstract

**Purpose:**

This review aims to explore and summarise current knowledge of indocyanine green (ICG) fluorescence application in metabolic and bariatric surgery (MBS) and assess its potential in improving patient safety.

**Methods:**

The review was prepared according to the Preferred Reporting Items for Systematic Reviews and Meta-Analyses (PRISMA) recommendations. Evidence from PubMed, ScienceDirect and Ovid MEDLINE databases was independently screened in October 2022. The primary information and outcomes were the type of fluorescence application with potential patient benefit and the complication rate. The secondary outcomes consisted of the kind of dye, the application protocol, and the equipment used.

**Results:**

Thirteen publications were included, with six prospective observational studies, five case reports and two retrospective cohort studies and involved a total of 424 patients. The publications were categorized into four groups based on the method of fluorescence application. Sixty-six percent of the cases used fluorescence for LSG, 32.3% used it for RYGB, 1.2% for revisional surgery, 0.2% during removal of an adjustable gastric band, and 0.2% for LSG combined with Rossetti fundoplication. ICG was used on its own in the majority of studies, although in one case, it was used in combination with methylene blue. ICG administration protocols varied significantly. Complications occurred in three patients (0.71%): leaks were diagnosed in two cases, and one patient required a blood transfusion.

**Conclusion:**

The most popular type of use is intraoperative assessment of the blood supply. ICG application may reduce the risk of complications, with potential benefits in detecting ischemia and leaks, searching for bleeding in areas inaccessible to endoscopy, and non-invasive hepatopathy evaluation.

## Introduction

For decades, metabolic and bariatric surgery (MBS) has been known as the most successful treatment option for obesity and related complications. The number of procedures performed worldwide increases year by year [1]. The recommendations of the American Society for Metabolic and Bariatric Surgery (ASMBS) and the International Federation for the Surgery of Obesity and Metabolic Disorders (IFSO) provide new eligibility criteria for surgery with a lower body mass index (BMI) in patients suffering from obesity. Therefore, the number of procedures performed may increase at a more rapid rate in the coming years [2]. Due to the potential increase in the number of metabolic and bariatric surgeries performed, the search for new solutions to keep the complication rate at the current level—or even lower it—becomes necessary. Apart from the rapid progress of advanced laparoscopic equipment, electrosurgical instruments and robotic surgery, the use of indocyanine green (ICG) seems to be a promising option with the possibility of reducing complication rates, which ICG has been shown to be effective at in other surgical fields, such as colorectal, upper GI and liver surgery [3–8].

This review aims to explore and summarise the current knowledge of the use of fluorescence in the field of MBS and assess ICG’s potential to reduce the risk of complications, improve patient safety and become a standard of care in surgical obesity treatment. The secondary aim is to examine current practice and know-how, including the types and dosing of fluorescent agents, detection time and equipment used.

## Methods

While preparing the manuscript, we followed the Preferred Reporting Items for Systematic Reviews and Meta-Analyses (PRISMA) statement recommendations [9]. We included papers on human studies of adult populations undergoing minimally invasive MBS with obesity treatment intent, with all types of intraoperative fluorescence use accepted. Publications in languages other than English, book chapters, descriptive reviews, non-abstract studies and non-full text studies were excluded, along with papers on open surgical procedures and procedures not aimed at body mass reduction. Publications that met the above criteria qualified for the analysis.

**Fig. 1:**
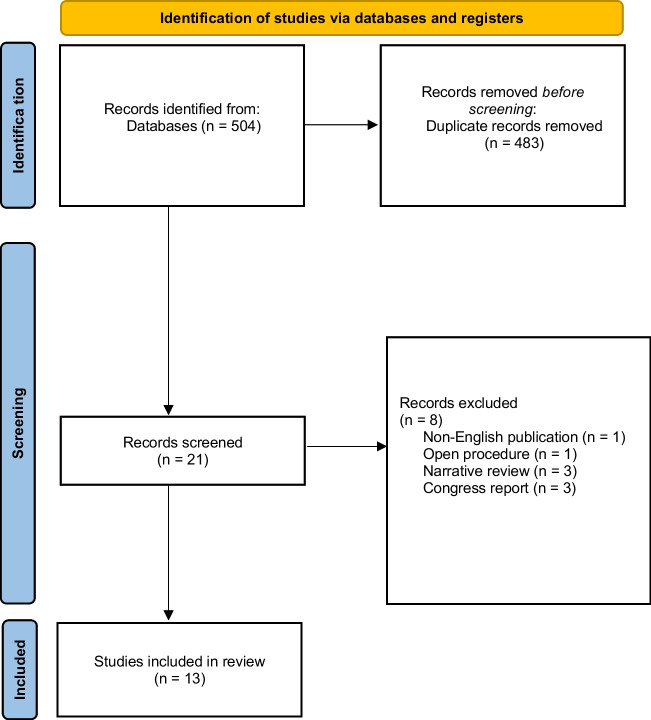
Flowchart of the study

Evidence from PubMed, ScienceDirect and Ovid MEDLINE databases was independently screened in October 2022 (last search 10/31/2022) by two authors (MW and MB). Various combinations of ‘fluorescence’, ‘indocyanine green’ and ‘ICG’ in combination with ‘bariatric surgery’, ‘metabolic surgery’ and the most common surgical procedures and their commonly known abbreviations—including ‘laparoscopic sleeve gastrectomy’ or ‘SG’, ‘Roux-en-Y Gastric Bypass’ or ‘RYGB’, and ‘One Anastomosis Gastric Bypass’ or ‘OAGB’—were used for the data search. Filters for human and adult populations were applied. Publications with abstracts that met the inclusion criteria of the intraoperative use of fluorescence in an adult population undergoing surgical treatment for obesity were qualified for the second verification stage. After removing duplicates, each study abstract was individually screened against the exclusion criteria by three authors (MW, MB, ND) before inclusion in the review (Fig. 1).

The primary information and outcomes we were looking for were the type of fluorescence application with potential patient benefit and the complication rate. The secondary outcomes included the kind of dye, the application protocol and the equipment used.

Only full-text publications were included in the analysis to reduce bias and ensure the best data quality. We did not apply any time restrictions, as this field is relatively new, and publications have been limited to the last few years. Abstract-only congress reports were excluded due to insufficient data. Descriptive reviews with data selection were not qualified for the synthesis.

## Results

A primary search of three different databases returned 504 publications. After removing 484 duplicated results, 21 studies met the inclusion criteria and were enrolled for further exploration. Eight of them were excluded for the following reasons: non-English language (*n* = 1) [10], open procedure (*n* = 1) [11], narrative reviews (*n* = 3) [12–14] and congress reports with the availability of the abstract only with insufficient data for inclusion (*n* = 3) [15–17]. This review included 13 publications, with six prospective observational studies, five case reports, and two retrospective cohort studies and involved a total of 424 patients.

Only four publications present the results of over 50 patients [18, 19, 27, 28], and the largest study group includes 95 patients [27]. Five studies were case reports that examined one patient each [23–26, 29]. The most commonly described application of fluorescence is the assessment of tissue blood supply during MBS, which was reported in nine studies [18–26]. Other related applications include leak testing [27, 28], assessment of gastrointestinal bleeding source [29] and non-invasive liver function check [30]. ICG was used in all studies. One study reported the simultaneous use of ICG and methylene blue [27]. Fluorescence was used in 280 (66.03%) patients for LSG [18–22, 24, 28, 30], 137 (32.32%) for RYGB [22, 25, 27–30], 5 (1.18%) for revisional surgery [22, 23], 1 (0.24%) for the removal of an adjustable gastric band (AGB) [30] and 1 (0.24%) for LSG combined with Rossetti fundoplication [26]. One hundred fifty-four (36.32%) surgeries were performed with the assistance of robotic technology [27, 28]. In the robotic surgery group, 89 RYGBs and 29 LSGs were performed. Complications occurred in three patients (0.71%): leaks were diagnosed in two cases [19, 24], and one patient required a blood transfusion [20].

### Risk of bias

The risk of bias was related to the small number of available publications, the mainly observational character of the studies, the different types of doses and methods of dye application and the various detection tools used.

The articles also lack basic information on ICG use, technology and patient characteristics. In all reviewed publications, the surgeon performed the fluorescence assessment subjectively. Another point is the lack of a standardised qualitative evaluation of ICG tissue saturation. The included papers provided no details on the physical and technical aspects of perfusion nor gave any information on parameters that would allow the description of normalisation or standardisation.

## Discussion

The publications included in this review of the literature in search of possible ICG applications in surgical obesity treatment and their potential benefits for patients were placed in four categories based on the method of application.

### Perfusion assessment

The main aim of this review was to assess adequate blood flow during surgery, as described in nine studies [18–26]. Balla et al. described a change in intraoperative strategy in two patients (15.4%) undergoing revisional surgery due to insufficient blood supply confirmed by ICG. Five months after the surgery, no complications were observed. However, the study group consisted of only 13 patients [22]. Di Furia et al. presented one case of a gastric leak in an SG group of 43 patients. In every single procedure, proper vascularisation was confirmed by ICG. The Gastrografin swallow test, assessing possible leaks, was negative in all cases. Nevertheless, the leak occurred on the fifth postoperative day and required endoscopic intervention [20]. Fifteen patients who underwent SG were retrospectively reviewed by Frattini et al. All gastric sleeves had sufficient intraoperative ICG perfusion. The resected part of the stomach was used to compare devascularisation. No complications were observed during the 2-month follow-up [21]. Vascular mapping to assess gastric blood supply before SG and gastric tube blood flow after sleeve formation was performed by Ortega et al. in 86 patients. The authors identified three main patterns of gastroesophageal blood supply with dominant right-sided flow ensured by left gastric, accessory gastric and hepatic arteries. No complications were observed in the study group [18]. Pavone et al. described a case of insufficient ICG perfusion, identified intraoperatively as a dark field in the proximal part of the gastric sleeve. This field was the source of the leak, which manifested 2 weeks after the surgery and required the endoscopic insertion of a gastric stent. The same author published a study of 82 patients who underwent SG with intraoperative ICG angiography. Perfusion was satisfactory in all cases. Intraoperative methylene blue and postoperative Gastrografin swallow tests were negative in all patients. Nevertheless, one patient developed a gastric leak and required the insertion of an endoscopic pigtail. The authors concluded that the leak rate was higher in the group of patients without ICG angiography (2.5%) than in the group of patients with intraoperative ICG (1.2%); however, they did not specify statistical significance [19, 24].

Unexpected intraoperative findings, modified surgical procedures, conversions or additional concurrent procedures might require perfusion assessment to ensure the proper functioning of the anastomoses. The most common use of fluorescence in MBS is for the assessment of the tissue blood supply. However, although the publications suggest that ICG may be helpful in this field, the available data might be significantly biased. Most publications describe small and retrospective studies without statistical analyses or metrics for the conducted interventions. There has been no standardised qualitative assessment of fluorescence in any publication. It seems necessary to improve the detection methods from a technical standpoint through adequate descriptions of the procedures to make them comparable. Prospective randomised controlled trials are needed for the conclusions.

### Leak testing

Two studies focused on using ICG for leak control during MBS. Both publications demonstrated the use of ICG injected through a bougie during robotic-assisted procedures. In a retrospective study of 59 patients, Kalmar et al. concluded that the ICG leak test had a comparable efficiency to intraoperative gastroscopy with 100% sensitivity and 98.28% specificity. Hagen et al. performed 95 robotic RYGB with intraoperative leak tests using a mix of methylene blue and ICG. ICG leakage was observed in 4.2% of cases, while the methylene blue test was negative. This analysis suggests that ICG detects small staple line defects more sensitively than the methylene blue test. The authors hypothesised that methylene blue might leak in too small amounts to be detected through a recording with a camera or the human eye without digital support in a dark operating field [27, 28].

The advantage of ICG over other leak-testing methods has not yet been established. The actual mechanism of ICG visibility with a simultaneous lack of MB visibility is unknown. Prospective randomised controlled trials with a standardised methodology and larger groups of patients are needed for further conclusions.

### Gastrointestinal bleeding mapping

A case report published by Copaescu et al. showed the interesting use of ICG. Of the source of bleeding after RYGB, the authors administered ICG preoperatively and after 22 min (the time needed for the dye’s elimination from the bloodstream). Using an infrared camera, the authors localised the extravasated ICG in the proximal part of the enzymatic loop. They concluded that the source of bleeding was located in the remnant. Such treatment allows for quick diagnosis and the stopping of gastrointestinal bleeding [29].

Based on this single case report, ICG might be an interesting option for further systematic exploration in cases that require locating the source of bleeding in the absence of lesions available for endoscopic examination.

### Non-invasive liver function testing

A liver biopsy is an invasive test that can be accompanied by complications, such as bowel perforation or bleeding. To determine the severity of non-alcoholic fatty liver disease (NAFLD), Danin et al. compared the liver biopsy results of 26 patients undergoing surgical treatment for obesity with an ICG clearance test performed on the same patients. The authors concluded that histological results correlate with the non-invasive evaluation of liver function using ICG. The dye may be used to screen for hepatopathy in the obese population [30].

The ability to measure liver function in a non-invasive manner is not associated with a surgical procedure per se. However, liver function in patients suffering from obesity is often disturbed, which may affect drug metabolism or cause coagulation disorders. In the authors’ opinion, the ability to non-invasively assess liver function without exposing the patient to biopsy-related complications, such as bleeding, may be helpful to the surgeon. However, this topic requires further research.

## Conclusion

This review presents various types of fluorescence applications in MBS. The most popular type of application is intraoperative assessment of the blood supply. ICG application may reduce the risk of complications and may potentially be beneficial for detecting ischemia and leaks, searching for bleeding in areas inaccessible to endoscopy and evaluating non-invasive hepatopathy. However, the scientific quality of the currently available evidence is low, and further controlled trials with proper methodologies that allow for comparisons are needed.
